# BRCA1/2 alterations and reversion mutations in the area of PARP inhibitors in high grade ovarian cancer: state of the art and forthcoming challenges

**DOI:** 10.3389/fonc.2024.1354427

**Published:** 2024-03-13

**Authors:** Laetitia Collet, Brunhilde Hanvic, Margherita Turinetto, Isabelle Treilleux, Nicolas Chopin, Olivia Le Saux, Isabelle Ray-Coquard

**Affiliations:** ^1^ Breast Cancer Translational Research Laboratory, Institut Jules Bordet, Hôpital Universitaire de Bruxelles (H.U.B), Université Libre de Bruxelles (ULB), Brussels, Belgium; ^2^ Medical Oncology Department, Centre Léon Bérard, Lyon, France; ^3^ University Claude Bernard Lyon 1, Lyon, France; ^4^ Department of Pathology, Centre Léon Bérard, Lyon, France; ^5^ Department of Surgery, Centre Léon Bérard, Lyon, France

**Keywords:** high grade ovarian cancer, BRCA1/2 mutation, reversion mutation, PARP inhibitor, resistance, homologous recombination deficiency

## Abstract

*BRCA1/2* genes are part of homologous recombination (HR) DNA repair pathways in charge of error-free double-strand break (DSB) repair. Loss-of-function mutations of *BRCA1/2* genes have been associated for a long time with breast and ovarian cancer hereditary syndrome. Recently, polyadenosine diphosphate–ribose polymerase inhibitors (PARPi) have revolutionized the therapeutic landscape of *BRCA1/2*-mutated tumors, especially of *BRCA1/2* high-grade serous ovarian cancer (HGSC), taking advantage of HR deficiency through the synthetic lethality concept. However, PARPi efficiency differs among patients, and most of them will develop resistance, particularly in the relapse setting. In the current proposal, we aim to review primary and secondary resistance to PARPi in HGSC owing to *BRCA1/2* alterations. Of note, as several mechanisms of primary or secondary resistance to PARPi have been described, *BRCA1/2* reversion mutations that restore HR pathways are by far the most reported. First, the type and location of the *BRCA1/2* primary mutation have been associated with PARPi and platinum-salt sensitivity and impact the probability of the occurrence and the type of secondary reversion mutation. Furthermore, the presence of multiple reversion mutations and the variation of allelic frequency under treatment underline the role of intratumor heterogeneity (ITH) in treatment resistance. Of note, circulating tumor DNA might help us to detect and characterize reversion mutations and ITH to finally refine the treatment strategy. Importantly, forthcoming therapeutic strategies, including combination with antiangiogenics or with targeted therapies, may help us delay and overcome PARPi resistance secondary to *BRCA1/2* reversion mutations. Also, progression despite PARPi therapy does not preclude PARPi rechallenge in selected patients.

## 1 Introduction

*BRCA1* and *BRCA2* are both tumor suppressor genes critical to maintaining genome integrity during DNA replication. They act within homologous recombination (HR) DNA repair pathways in charge of error-free double-strand break (DSB) repair using undamaged sister chromatin as a template (1), unlike the error-prone repair of DNA through nonhomologous end-joining (NHEJ) repair pathways. However, *BRCA1* and *BRCA2* are involved at different levels during DSB repair. BRCA1 first promotes 5′ end resection of the DSB and then acts in association with PALB2 and BRCA2 to recruit RAD51 at the DNA damage site. Furthermore, BRCA1/2 is also in charge of replication fork protection under replicative stress, and BRCA1 is also implicated in cell-cycle checkpoint activation and acts on the NHEJ repair pathways via the RAD50/MRE11/NBS1 complex (2, 3). Germline mutations in these genes have been associated for a long time with breast and ovarian cancer hereditary syndrome, and more recently, pancreas and prostate cancers have also been linked to *BRCA1/2*-mutated cancer spectrum (4). BRCA1/2 germline mutation also confers better outcomes, especially in ovarian cancer patients (5). Over the past decade, polyadenosine diphosphate–ribose polymerase inhibitors (PARPi) have emerged as a major therapeutic breakthrough for patients with *BRCA1/2*-mutated tumors and more widely for patients with HR-deficient tumors (6–9). PARPi efficacy is mainly based on synthetic lethality. PARPi prevents the repair of single-strand breaks (SSBs) occurring during phase S of the cell cycle, therefore promoting the DSBs, owing in part to PARP trapping and the collapse of the replication fork (10). In the case of HR deficiency (HRD), such as a loss of function mutation in the *BRCA1/2* gene, the accumulation of unrepaired DBSs leads to genomic instability and cancer cell death. High-grade serous ovarian cancer (HGSC) is the second-most lethal gynecological cancer worldwide (11). In total, 30% of HGSC harbor somatic or germline *BRCA1/2* loss-of-function mutations, and about 50% are associated with HRD (12). Platinum-based chemotherapy and debulking surgery are the long-standing cornerstones of therapeutic strategy (13), and HGSC patients have been the first to show a benefit of PARPi over other *BRCA1/2*-associated cancers. PARPi efficacy was demonstrated first in platinum-sensitive relapse ovarian cancers. SOLO2 trial showed a clinically meaningful while not statistically significant benefit in overall survival (OS) in *BRCA1/2*-mutated patients with olaparib maintenance (14). Thereafter, niraparib as a second-line maintenance therapy demonstrated an advantage in progression-free survival (PFS) in all comers, including *BRCA1/2* wild-type patients, but failed to demonstrate an OS benefit as presented at the Society of Gyneco-oncology 2023 (15) that advocate the use of PARPi in earlier setting. The SOLO1 trial was the first phase III clinical trial that demonstrated a benefit in PFS with olaparib maintenance in *BRCA1/2* mutant newly diagnosed HGSC patients (16). Thereafter, niraparib and rucaparib also showed a survival benefit in maintenance therapy in all comers, although *BRCA1/2*-mutated patients, followed by patients with HRD tumors, still derived the greatest benefit from PARPi (17, 18). Of note, updated OS in the SOLO1 trial showed that 67% of *BRCA1/2* mutant patients are still alive after 7 years of follow-up and 45% did not even receive subsequent therapy, giving hope for a potential cure (16). More recently, PAOLA-1 has been the first clinical trial to demonstrate the benefit of olaparib in association with bevacizumab in first-line maintenance therapy. An increase in OS was observed in *BRCA1/2*-mutated patients and in the HRD population (6), suggesting that maintenance combination therapy might increase the benefit of PARPi in these patients. However, despite these major advances, most patients will relapse and die of drug-resistant ovarian cancers. Three main mechanisms of PARPi resistance have been reported, encompassing resistance related to the drug target, including *PARP1* mutations or upregulation of drug efflux pumps, restoration of HR pathway, and restoration of fork stability (19). Whereas the majority of PARPi resistance mechanisms have been extensively described *in vitro* (20–24), only a few have been reported in patients. Among them, restoration of the HR pathway as a consequence of *BRCA1/2* reversion mutations (25, 26) is the most well-known mechanism of resistance. First described in cancer cell lines and patient-derived xenografts (PDX) models, reversion mutations have been identified and are currently increasingly studied in patients enrolled in clinical trials (27, 28). Moreover, beyond the role of *BRCA1/2* reversion mutations in PARPi resistance, the type and location of the original loss of function mutation have been recently associated with primary PARPi sensitivity (29). In the current proposal, we aim to review primary and secondary resistance to PARPi in HGSC owing to *BRCA1/2* alterations. After describing *BRCA1/2* alterations and reversion mutations that impact PARPi sensitivity and efficiency, we will further analyze the role of circulating tumor DNA (ctDNA) sequencing to detect them and improve therapeutic strategy. Finally, we will analyze forthcoming therapeutic strategies to overcome PARPi resistance that occurs along with *BRCA1/2* reversion mutations.

## 2 Primary resistance to PARPi

### 2.1 PARP inhibitors sensitivity according to *BRCA1* and *BRCA2* mutation type

*BRCA1* and *BRCA2* loss-of-function mutations are the most well-recognized predictive biomarkers of response to PARPi (6, 17). *BRCA1/2* genes are characterized by different functional domains (2). *BRCA2* has three main functional domains, namely the RAD51-binding domain (RAD51-BD), a C-terminal DNA-binding domain (DBD), and the BRC or TR2 domains that interact with RAD51 filaments. All are involved in HR pathways (2). Moreover, *BRCA1* functional domains include a highly conserved N-terminal Really Interesting New Gene (RING), a DBD, and a C-terminal domain of *BRCA1* (BRCT). While they are all involved in DNA repair, BRCT is also implicated in the cell cycle through G2/M and S-phase checkpoint. Consequently, some studies reported distinct outcomes in *BRCA1* or *BRCA2* loss of function mutation carriers treated with PARPi. A *post-hoc* analysis of study 19 and ARIEL2 trials showed better outcomes in *BRCA2* mutation carriers, with olaparib and rucaparib, respectively (30, 31). Of note, patients with *BRCA1* promoter hypermethylation derived benefit from rucaparib, but no one was observed among long-term responders, and two experienced poor outcomes despite olaparib therapy in study 19 (30). The location and type of mutation also confer distinct sensitivity to PARPi. *In vitro* studies showed that tumors with mutation in exon 11 of *BRCA1* are less sensitive to PARPi than those with mutation outside the exon 11 (32). Moreover, deletion in DBD of *BRCA2* increases olaparib and cisplatin sensitivity of engineered cell lines while deletion within the C-terminal domain retains partial HR activity and confers less sensitivity to DNA-damaging agents (33). Recently, ancillary analysis from the PAOLA-1 phase III clinical trial (29), assessing olaparib and bevacizumab first-line maintenance therapy, was the first large effort to assess the impact of the location of *BRCA1/2* mutations on PARPi sensitivity in patients (6). Labidi-Galy and colleagues analyzed the location of *BRCA1/2* mutation in 233 out of 806 randomized patients. Interestingly, they found that patients with *BRCA1/2* mutation involving exon 11 derived greater benefit from the addition of olaparib compared to patients with mutation outside the exon 11 (HR = 0.2 [95% CI = 0.11–0.36] and HR = 0.41 [95% CI, 0.22–0.75], respectively) (29), as well as those with mutations in DBD of *BRCA1* (HR = 0.08 [95% CI = 0.02–0.28]). These results are, however, surprising since previous studies reported worse outcomes and sensitivity to PARPi and platinum in cell lines with *BRCA1* mutation inside versus outside exon 11 due to the presence of a hypomorphic BRCA1 protein (32). Nevertheless, the median PFS (mPFS) of 16 months in these patients in the PAOLA-1 trial suggests lower platinum sensitivity (29) but still deserves further analyses, specifically regarding cross-resistance between platinum and PARPi and BRCA1 protein functionality. In *BRCA2* mutation carriers, whereas patients with mutation in *RAD51*-BD had a significantly longer PFS with the addition of olaparib (HR = 0.31 [95% CI = 0.11–0.92]), those with mutation in DBD had an excellent outcome in both arms (24 months PFS of 90% and 100% with and without olaparib, respectively), consistent with *in vitro* studies showing a substantial PARPi and platinum sensitivity in DBD *BRCA2*-mutated cell lines (33) and very rare reversion mutations in that domain (25). Of note, subgroup analysis from the PAOLA-1 and SOLO1 trials showed that *BRCA1* mutation carriers derived greater benefit from PARPi (HR = 0.40 [95% CI = 0.29–0.56] and HR = 0.20 [95% CI = 0.10–0.38] for PFS in *BRCA2* and *BRCA1* carriers, respectively, in SOLO1 and HR = 0.5 [95% CI = 0.34–0.73] and HR = 0.2 [95% CI = 0.11–0.39]), respectively, for PFS in PAOLA-1) (29, 34). Altogether, these data explain, in part, different outcomes in patients with *BRCA1* or *BRCA2* mutations treated with PARPi, with some patients experiencing a very long response and a potential cure, while others have quick progression despite a *BRCA1/2* mutation or HRD profile.

### 2.2 Hypomorphic BRCA1 protein and primary PARP inhibitor resistance

A few *BRCA1* mutations lead to a hypomorphic BRCA1 protein that retains HR activity and promotes PARPi resistance. For instance, a frameshift mutation in exon 11 leads to the BRCA1-Δ11q splice variant transcript and a BRCA1 protein with HR activity measured by RAD51 γ-irradiation-induced foci formation *in vitro*. Even though BRCA1-Δ11q HR activity is inferior to BRCA full-length, this drives partial PARPi resistance (32). Importantly, this variant has been found in postprogression tumor samples from patients previously treated with rucaparib in the ARIEL2 trial (35). Similarly, alterations in the RING domain of *BRCA1*, like BRCA1185delAG or BRCA1C61G, also translate into a hypomorphic RING-less BRCA1 protein with a partial DNA damage response that decreases sensitivity to PARPi and platinum therapy. Mice with a hypomorphic RING-less BRCA1 protein become rapidly resistant to PARPi and platinum without acquiring reversion mutations (36, 37). Altogether, these data showed that the BRCA1 RING domain and exon 11 are dispensable, to some extent, for HR activity. In contrast, hypomorphic BRCA1 proteins that lack domains in the C-terminal region might need the alteration of additional pathways to acquire PARPi resistance. For instance, a stop codon in the coiled-coil domain of *BRCA1* results in a hypomorphic protein that acts downstream of end resection and brings PARPi resistance only in a *53BP1* gene knockout mouse model (38). In addition, the BRCT-less BRCA1 chimeric protein is usually destroyed and therefore needs to be stabilized by HSP-90 to escape proteasome degradation, interact with the PALB2-BRCA2-RAD51 complex, and drive PARPi and platinum resistance (39). Even if less described, some hypomorphic BRCA2 proteins have also been reported. Although no relationship between PARPi and platinum sensitivity was reported, *BRCA2* C-terminal DNA-binding domain deletion but conserved BRC repeat motifs allowing complex with RAD51 and HR activity has been described *in vitro* (40). Another *in vitro* study also showed that duplication of the *BRCA2* mutant allele lacking the DBD along with the overexpression of truncated protein led to PARPi resistant cell lines. However PARPi resistance requires a Disruptor Of Telomeric silencing 1-Like (DOT1L) to interact with BRCA2 truncated protein, suggesting that hypomorphic BRCA2 is not sufficient by itself to promote PARPi resistance and needs an additional mechanism of HR recovery (41).

### 2.3 Acquired reversion mutations under platinum-based chemotherapy and crossresistance with PARPi

It is now well-admitted that, beyond *BRCA1/2* mutation and HRD status, patients responding to platinum-based chemotherapy are the ones who benefit most from PARPi (42). Furthermore, platinum resistance confers a poor benefit of subsequent PARP inhibition. Recent data also showed a poorer survival and overall response rate (ORR) with platinum rechallenge after PARPi maintenance therapy, regardless of platinum-free interval (PFI) and *BRCA1* or *BRCA2* mutations (43–46). Altogether, these data suggest that platinum and PARPi resistance mechanisms partially overlap. *BRCA1/2* reversion mutations after platinum-based chemotherapy have been reported to occur in 20% to 40% of platinum-resistant or refractory diseases (26, 47, 48), while in less than 5% in platinum-sensitive settings (27, 28, 48) and jeopardize primary PARPi efficacy. Of note, the occurrence of reversion mutations in platinum-sensitive diseases also highlights the limit of the platinum-free interval as the only definition of platinum sensitivity. Indeed, *post-hoc* analysis of OlyimpiAD (8), SOLO3 (49), and LIGHT (50) trials assessing olaparib in *BRCA1/2* mutant breast cancers and recurrent platinum-sensitive ovarian cancer patients showed that 4% (*N* = 4/114) and 3% (*N* = 4/130) of patients with breast and ovarian cancers, respectively, already harbored *BRCA1/2* reversion mutations before PARPi, despite a platinum-sensitive disease as defined with PFI (28). Moreover, ancillary analysis from the ARIEL2 study assessing rucaparib in platinum-resistant and platinum-sensitive patients reported enrichment of reversion mutations in platinum-resistant or refractory diseases (13% and 18%, respectively) compared to platinum-sensitive diseases (2%) (31). Of note, for three patients, including one with platinum-refractory disease and two patients with platinum-resistant diseases, five, four, and eight reversion mutations, respectively, were detected using ctDNA. Patients with *BRCA1/2* reversion mutations before PARPi had a poorer PFS with rucaparib compared to patients without reversion mutations (mPFS 9 months and 1.8 months, respectively, HR = 0.12 [95% CI = 0.05–0.26]; *p* < 0.0001). Interestingly, among patients with platinum-resistant or refractory disease, those with a reversion mutation had a poorer prognosis (mPFS 7.3 months and 1.7 months, respectively, HR = 0.16 [95% CI = 0.07–0.42]; *p* < 0.0001, in patients without and with BRCA1/2 reversion mutation) (27), suggesting that we should refine the prognostic groups based on molecular characteristics. Similarly, Norquist et al. reported a reversion mutation in 46% of platinum-resistant HGSC patients versus only 5% in platinum-sensitive tumors (48). Among patients with platinum-resistant ovarian cancers and mutation reversion, two out of three were resistant to subsequent PARPi, whereas, surprisingly, one patient still derived benefit from PARPi and experienced a partial response despite *BRCA1* reversion mutation to wild type and a platinum-resistant disease. Moreover, three patients with platinum-resistant disease did not have reversion mutations and experienced a partial or complete response with PARPi (51). Thus, although reversion mutations usually confer crossresistance between platinum and PARPi, some might specifically drive platinum resistance while retaining PARPi sensitivity, and some mechanisms of platinum resistance might be independent of reversion mutations and consequently not overlap with PARPi resistance. Reversion mutation types and underlying mechanisms might also help to predict PARPi resistance in patients with reversion mutations occurring under platinum-based chemotherapy. As more extensively described below, types of reversion mutation usually overlap with those that appear under PARPi or platinum-based chemotherapy, and microhomology end joining (MMEJ) is involved in both situations (26, 47). These strengthen the idea that most of the reversion mutations might confer PARPi and platinum crossresistance. However, larger deletions are enriched in reversion mutations affecting the *BRCA2* gene after PARPi compared to platinum therapy (26), suggesting that some mechanisms could also be drug-specific.

## 3 Secondary resistance to PARPi

If *BRCA1/2* mutations or HRD tumors are strongly vulnerable to PARP inhibition, providing survival improvement in clinical practice, most patients will develop resistance. Among the different mechanisms of resistance reported, the occurrence of the *BRCA1/2* reversion mutation is the most recognized and described to date.

### 3.1 *BRCA1/2* acquired reversion mutations under PARP inhibition

**Figure 1:**
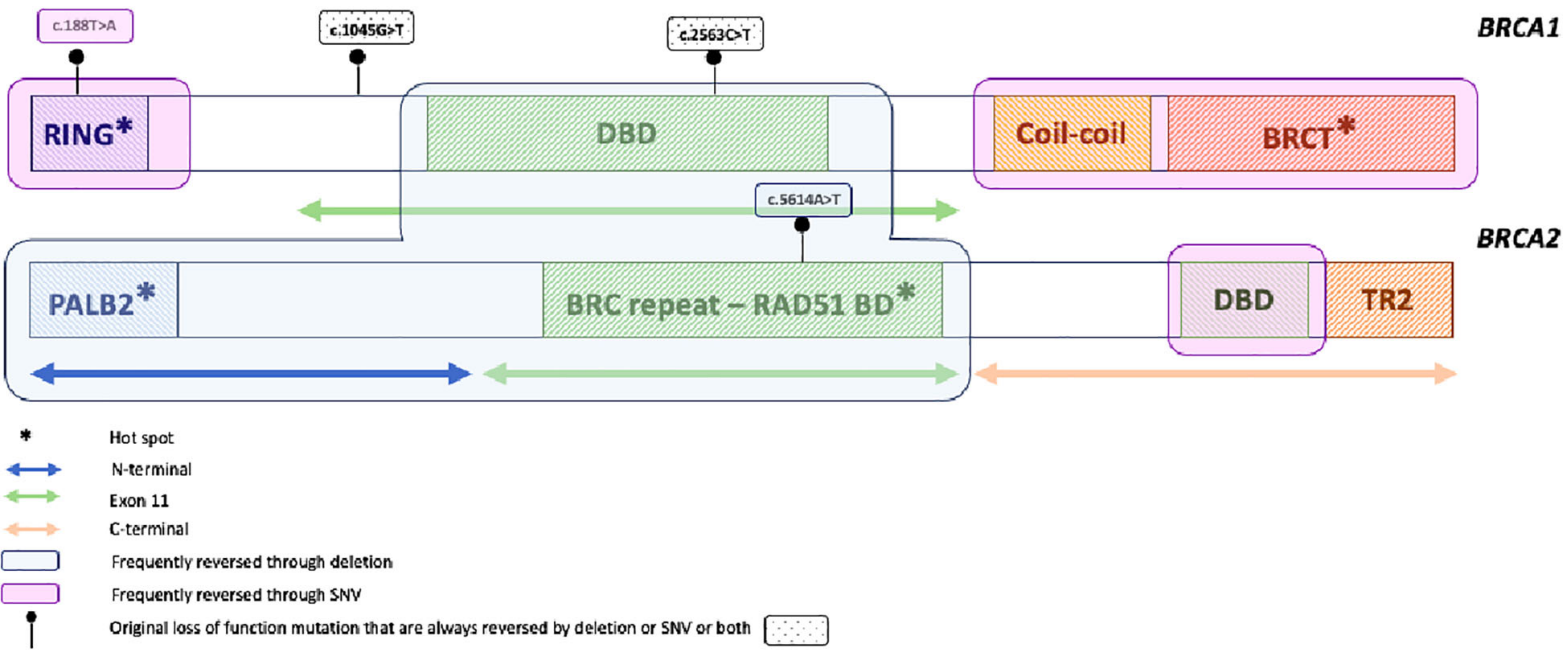
Pattern of reversion mutation in the *BRCA1* and *BRCA2* genes. *: hot spot mutation.

The most reported mechanism of HR restoration under PARPi is the reversion mutation of the *BRCA1/2* gene, which restores the expression of *BRCA1/2* functional protein and HR pathways. *BRCA1/2* reversion mutations encompass second-site deletion or insertion and the in-frame deletion of the original mutation, both restoring the open reading frame, and the true reversion mutation, namely “mutation reversion to wild type”, restoring the wild-type gene sequence (25). *BRCA1/2* reversion mutations have first been studied using *in vitro* cancer cell lines, demonstrating the restoration of open reading frame and HR activity that confer platinum and PARPi crossresistance (52, 53). Later, reversion mutations were studied on PDX models treated with DNA-damaging agents. Under cisplatin or olaparib treatments, Ter Brugge and colleagues described a *BRCA1/2* original mutation deletion that restored the open reading frame and, thus, the production of *BRCA1* functional protein. They also described the loss of methylation of the *BRCA1* promotor as well as the fusion of *BRCA1* with a heterologous promotor that allows for transcription of hypermethylated *BRCA1* and confers PARPi and platinum resistance in PDX model with *BRCA1* promotor hypermethylation (54). Next, using samples from PARPi-resistant patients, the reversion mutation under PARP inhibitors was transferred into the clinic. Interestingly, several teams report different clinical cases involving breast (55, 56), ovarian (35, 57), pancreatic (58), and prostate cancers (59), highlighting that PARPi resistance mechanisms would be shared between cancers of the *BRCA1/2* spectrum. *BRCA1/2* reversion frequency under PARPi accounts for 10% to 40%, depending on the studies (26–28, 47, 60, 61), and can occur on *BRCA1/2* somatic or germline original mutants and in platinum-sensitive or resistant settings. In ARIEL2 *post-hoc* analysis (31), reversion mutations that appear under rucaparib were detected on cfDNA samples for eight out of 78 patients (10%), regardless of platinum sensitivity, and were detected at a median of 3.4 months before radiological progression. Interestingly, among the three patients who harbored multiple reversion mutations acquired before PARP inhibition, likely secondary to previous platinum-based chemotherapy, the mutation allele frequency (MAF) of the different reversion mutations changed under PARPi pressure, suggesting that some reversion mutations may preferentially drive PARPi resistance while others might retain, to some extent, PARPi sensitivity. More recently, Pettitt et al. and Tobalina et al. reviewed all published clinical cases reporting *BRCA1/2* reversion mutations in cancers of the *BRCA1/2* spectrum, namely ovarian, breast, prostate, and pancreatic cancers (25, 26). Pettitt et al. also included relevant studies from cell lines and PDX models (25). In both studies, most patients were treated for ovarian cancer, which was expected regarding the extensive use of platinum and PARPi in this tumor type. They reveal that most of the reversion mutations are unique, reported as “single-patient mutations”, except for the stronger propensity of true-reversion mutation to wild-type recurrent reversion mutations (25). Reversion mutations occur more frequently in *BRCA2* than in *BRCA1* genes. Within each gene, they also identified regions more prone to be impacted by reversion mutations, namely “hotspot” regions, and those called desert regions with very few reversion events (**Figure 1**). Within *BRCA2*, reversion mutations were frequently observed in N-terminal or RAD51BD domains, namely hotspot, while they were relatively rare in the highly conserved C-terminal region, encompassing the DBD domain, namely “a desert” (25, 26) (**Figure 1**). Of note, reversion mutations occurring in this region were a true reversion to wild-type or missense reversion mutations, whereas deletions were more frequently observed in the hotspot region. In *BRCA1*, BRCT and RING domains are found to be the hotspots (26). The type of original loss-of-function alteration also impacts the likelihood of being reversed. Reversion of original frameshift deletion or insertion pathogenic mutations is more frequent that reversion of original missense or splice site mutations, which are relatively rare (25–27). Secondary mutations themselves are mainly deletions, with a larger size observed in the *BRCA2* gene. Larger deletions are especially seen in exon 11 of both *BRCA1* and *BRCA2*, highlighting less conserved and dispensable sequences with regard to *BRCA* function, as previously described (32). However, more substitution or reversion to wild type occurs in *BRCA1*, suggesting that the mechanism of reversion mutation in the *BRCA1* gene might arise from a larger range of DNA repair mechanisms. Thus, the type of secondary mutation depends on the location and type of primary mutation (25, 26). While the mechanism of reversion mutation is not fully understood, the identification of DNA sequence microhomology at the end of the site of reversion mutation highlights that MMEJ may be responsible for, at least a part of reversion mutations. The detection of microhomology sequences is more frequently reported in *BRCA2* than *BRCA1*, although surround reversion mutations occur in more than half of the cases and are longer in *BRCA2* (25, 26). Notably, whereas reversion mutations seem to be more frequent in the *BRCA2* gene, *BRCA2* mutant carriers do not have a worse outcome, and the opposite has even been reported. As previously described, reversion mutations are often large deletions that could produce hypomorphic proteins. As mentioned above, while BRCA1 hypomorphic protein has been reported as PARPi primary resistance mechanism (32), hypomorphic *BRCA2* protein might need additional alteration to drive HR restoration (40, 41). Moreover, longer sequences of microhomology and secondary deletion or insertion, more frequent in *BRCA2*, introduce novel amino-acid sequences and may constitute neoantigens and drive immunogenic antitumor activity (25). However, we should be aware that these data are only hypothesis-generating and should be further explored. Beyond reversion mutations, heterozygosity also plays a role in PARPi efficacy. Lheureux et al. reported upregulation of *BRCA1* and *BRCA2* along with gene copy number gain as a mechanism of resistance in two patients who experienced a very long response under PARPi (57). Of note, no one had germline *BRCA1/2* loss of function at baseline. Similarly, loss of homozygosity or complete loss of *BRCA1* promotor hypermethylation in PDX models of *BRCA1*-methylated HGSC is also associated with PARPi resistance, demonstrating that hypermethylation of all *BRCA1* copies are required to predict response to PARPi (54, 62). Thus, deciphering *BRCA1/2* mutation reversion might help tailor the patient’s follow-up and treatment strategy. Patients with *BRCA2* mutations in DBD have an excellent prognosis and a low risk of reversion mutation occurrence, while patients with *BRCA2* mutations in the N-terminal domain or in BRCT or RING domain of *BRCA1* should be closely monitored. Moreover, beyond *BRCA1/2* mutations, copy number and gene expression should be taken into consideration to estimate the probability of both response and resistance.

### 3.2 Reversion mutation in other HR-related genes

The use of gene panels searching for mutations in HR-related genes other than BRCA1/2 failed to predict response to PARPi in ovarian cancers (63), limiting such use in clinical practice. However, loss of function of specific HR-related genes, such as *RAD51C* and *RAD51D*, has still been associated with PARPi response (31, 64), and reversion mutations have also been reported in this setting. *Post-hoc* analysis from ARIEL2 part I study assessing rucaparib in platinum-sensitive HGSC patients showed that secondary mutations in *RAD51C* and *RAD51D* that restore the open reading frame allow the production of functional protein and resistance to PARPi, further validated *in vitro* (35). More specifically, one patient harbored four distinct *RAD51C* reversion mutations in a postprogression sample, all responsible for multidrug resistance in culture cell lines, including platinum (cisplatin and carboplatin) and PARPi (rucaparib, olaparib, veliparib, talazoparib, and niraparib). Interestingly, different adjacent core biopsies collected to generate PDX models revealed that one core predominantly contained one specific reversion mutation (c.574_577delinsGGCG mutation) and underlined substantial intratumor heterogeneity and the emergence of resistant clones under PARPi selective pressure. In another patient, a *RAD51D* reversion mutation was also reported, but only within a splenic lesion that was progressing on rucaparib, while it was not found in liver metastasis still responding to rucaparib. In addition, loss of *RAD51C* promoter methylation has also been reported to confer PARPi resistance (65), with loss of methylation in a single copy being sufficient to cause PARPi resistance (66), as was previously shown in *BRCA1*-hypermethylated ovarian cancers. In the EVOLVE study, authors also described a reversion mutation of *RAD51B* as well as an overexpression of *RAD51C* in a second patient after olaparib failure (61). *RAD51* amplification has also been reported in PDX models (67). Beyond *RAD51*, the *PALB2* reversion mutation has also been described *in vitro* (68) and then reported in patients treated with olaparib for prostate cancer (69, 70). Therefore, reversion mutation that drives PARPi resistance is not restricted to *BRCA1/2* genes and should be considered among biomarkers of response and resistance to PARPi.

### 3.3 The role of circulating tumor DNA to detect reversion mutations

**Figure 2:**
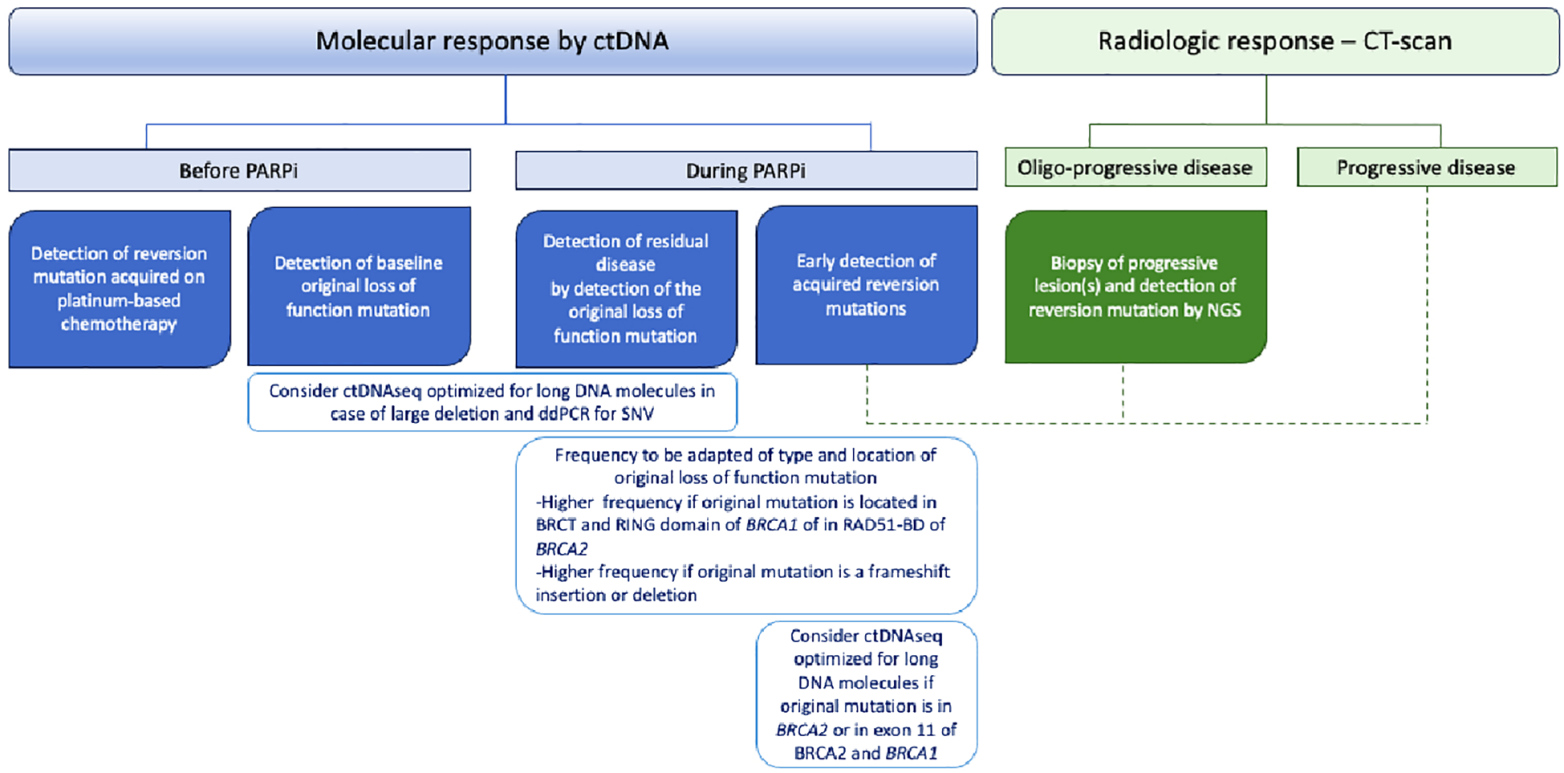
Potential roles of circulating tumor DNA (ctDNA) in BRCA1/2-mutated high-grade serous ovarian cancer patients.

Liquid biopsies and ctDNA analysis have been increasingly studied in the past few years (71). In ovarian cancer, several studies described reversion mutations detected on ctDNA with high sensitivity and specificity and a strong correlation with mutations found within tumors (27, 60). In a *post-hoc* analysis of the ARIEL2 trial, cell-free DNA (cfDNA) was able to detect ctDNA in 81% and 96% of pre- and posttreatment samples, respectively (27). Similarly, Weigelt and colleagues also detected ctDNA in 95% of blood samples from patients with ovarian cancers (47), and another team reported a sensitivity and specificity of 60% and 90%, respectively, to detect reversion mutations in ctDNA (60). In addition to the advantage of being minimally invasive, one of the major interests of liquid biopsy is to detect intratumor heterogeneity, which allows for a more global tumor assessment. Indeed, distinct reversion mutations are found within tumor subclones that arise with disease evolution and PARPi selective pressure. For instance, Barber et al. reported a single *BRCA2* reversion mutation occurring in a metastatic lymph node that was the only site of progression. No reversion mutation was found in the primary tumor or peritoneum metastasis (56). Another particularly notable example is from Patch et al., who reported 12 distinct *BRCA2* reversion mutations using multiple metastatic lesion collections from an autopsy patient. Some reversion mutations were shared between several metastatic sites, while others were site-specific and therefore could be used to describe cancer phylogeny (12). Therefore, in clinical practice, ctDNA might help to detect a larger number of reversion mutations stemming from different tumor subclones that would not have been detected with a single tumor solid biopsy. Lin et al., in an ancillary analysis from the ARIEL2 trial, reported additional reversion mutations detected on ctDNA compared to NGS of solid tumor tissue (27). Furthermore, MAF variation of the reversion mutations acquired on platinum-based chemotherapy under PARPi highlighted subclone expansion under PARPi pressure. Multiple reversion mutations were also more frequently found in ctDNA than in paired tumor samples in the prospective analysis from Weigelt et al. on 24 HGSC patients (47). Importantly, a patient with reversion mutations of *BRCA1* found in ctDNA was detected neither in primary tumors nor in peritoneum metastasis samples. Of note, Christie and colleagues reported the reversion mutations from paired ctDNA and ascite samples of 30 retrospectively selected HGSC patients. Unlike previously described, more reversion mutations were detected in ascite samples than in ctDNA, which is consistent with the highly conserved somatic mutation described in ascitic fluid (72). Another opportunity that ctDNA offers is the detection of reversion mutations occurring during the course of the disease in a “real-time” manner. Jacob and colleagues experimented with this concept in a patient treated for an advanced HGSC. By collecting the ctDNA at several time points, they described several reversion mutations that emerged during the course of the disease along with successive treatments (73). Moreover, some studies showed that ctDNA may detect reversion mutations before radiologic or clinical progression (27, 47, 60), although the benefit of an earlier modification of treatment strategy is still unanswered. Thus, even more promising would be the use of ctDNA as a surrogate biomarker of response to PARPi. In a recent prospective phase I clinical trial assessing camonsertib, an ATR inhibitor, in 120 patients with solid tumors and alteration in DNA damage repair genes, the investigators collected ctDNA at baseline and at each cycle of treatment to perform targeted sequencing. They defined molecular response as a 50% decline in the mean variant allele frequency (MAF) of the original somatic variant. A total of 54% (*N* = 7/13) of evaluable HGSC patients and 53% (*N* = 15/28) of patients with *BRCA1/2* mutations had a molecular response. Although not statistically significant, the molecular response was more frequently observed in patients with clinical benefit versus not (66% vs. 25% and 69% vs. 40% in ovarian cancer and *BRCA1/2* mutant subgroups, respectively) (74). However, ctDNA analysis does not come without drawbacks, and technical specificity might miss some alterations. For instance, in the ARIEL 2 trial, a large deletion of *BRCA1* that restored the open reading frame in a platinum-resistant patient was detected in the tumor sample but not in ctDNA (27). Thus, the short cfDNA fragments and short paired-end read sequencing usually used for ctDNA sequencing should be overcome in the future to detect specific alterations, such as large deletions, by the implementation of ctDNA sequencing new cutting-edge technologies (75, 76). Thus, ctDNA should be used in the near future to assess the molecular response to PARPi as well as to detect the earlier occurrence of the *BRCA1/2* reversion mutation. Of note, the frequency of ctDNA assessment should be tailored to the risk of progression under PARPi considering, among others, the original loss of function mutation of *BRCA1/2*, with a higher risk of reversion mutation occurrence and earlier recurrence if original mutation is a frameshift deletion or insertion or is within the BRCT or RING domain of *BRCA1* and in RAD51-BD of *BRCA2* (**Figure 2**).

## 4 Overcoming PARP inhibitor resistance in the setting of *BRCA1/2* reversion mutation

The understanding of PARPi resistance and the mechanisms of *BRCA1/2* reversion mutations paves the way for forthcoming new therapeutic strategies. Combination therapy with PARP inhibitors might overcome or delay PARPi resistance, and surgery still plays a major role. Various combinations are currently being explored encompassing the antiangiogenic-, POLθ-, PI3K/AKT/mTOR-, or RAS/RAF/MEK-targeted therapies (**Tables 1**, **2**).

**Table 1:** Clinical trials of PARPi combination in ovarian cancer recruiting/not yet recruiting.

Trial	Phase	Treatment	Study population
Anti-VEGF			
NCT02484404	I/II	Durvalumab +/− olaparib +/− cediranib	Pan tumor, cohort 1: advanced/recurrent ovarian cancer PARPi pretreated allowed
PIK3CA inhibitor			
NCT04729387 (EPIK-O)	III	Olaparib + alpelisib vs. standard chemotherapy	Resistant/refractory platinum ovarian cancer, without germline BRCA mutation. PARPi pretreated allowed
NCT05564377	II	Olaparib + alpelisib	Cohort 3: PARPi-resistant ovarian cancer
BET inhibitor			
NCT05071937	II	Talazoparib + ZEN003694	Recurrent epithelial ovarian cancer. PARPi pretreated
ATR inhibitor			
NCT02264678	I/Ib	Ceralasertib + olaparib	Module 2 (B5): patient with HRD recurrent platinum-sensitive ovarian cancer who progressed on PARPi Module 5: recurrent platinum-sensitive ovarian cancer PARPi pretreated
WEE1 inhibitor			
NCT03579316	II	Adavosertib +/− olaparib	Recurrent ovarian cancer PARPi pretreated allowed

PARPi, polyadenosine diphosphate–ribose polymerase inhibitors; HRD, homologous recombination deficiency.

**Table 2:** Clinical trials of PARPi combination in ovarian cancer with results.

Trial	Phase	Treatments	Study population	Results	Grade III/IV Aes > 10%	Reference
VEGF inhibitor						
NCT03117933 (OCTOVA)	Phase II randomized	Olaparib +/− cediranib vs. weekly paclitaxel	Recurrent platinum-resistant ovarian cancer Prior PARPi allowed (*n* = 139)	- O + C vs. O: HR = 0.70; 60% CI = 0.57, 0.86; *p* = 0.08 - Paclitaxel vs. O: HR = 0.97; 60% CI = 0.79, 1.19; *p* = 0.55	–	Nicum et al. (77)
NCT02681237 (EVOLVE)	Phase II	Olaparib + cediranib	Progression on PARPi for recurrent ovarian cancer (*n* = 34)	ORR 3/34 (9%) SD 20/34 (59%)	Diarrhea (12%)	Lheureux et al. (61)
PIK3CA inhibitor						
NCT01623349	Phase I	Olaparib + alpelisib	Recurrent platinum-resistant ovarian cancer Prior PARPi allowed (*n* = 28)	ORR 10/28 (36%) SD 14/28 (50%)	Hyperglycemia (15%)	Konstantinopoulos et al. (78)
ATR inhibitor						
NCT03462342 (CAPRI)	Phase II	Olaparib + ceralasertib	Recurrent platinum-sensitive ovarian cancer PARPi pretreated (*n* = 12)	ORR 6/12 (50%)	Anemia (15%) Thrombocytopenia (23%)	Wethington et al. (79)
NCT02576444 (OLAPCO)	Phase II	Olaparib + ceralasertib	PARPi-resistant ovarian cancer with HR gene alteration (*n* = 7)	ORR 1/7 (14%) SD 5/7 (71%)	1 patient: grade 3 anemia and grade 4 neutropenia	Mahdi et al. (45)
WEE1						
NCT03579316 (EFFORT)	Phase II randomized	Adavosertib +/− olaparib	Recurrent ovarian, fallopian tube, or primary peritoneal cancer with progression disease on PARPi (*n* = 80)	- Cohort A: ORR = 23% PFS = 5.5 months - A+ O = 29% PFS = 6.8 months	Cohort A: neutropenia (13%); thrombocytopenia (10%) Cohort A/O: thrombocytopenia (20%); neutropenia (15%); diarrhea (12%); fatigue (12%); anemia (10%)	Westin et al. (80)
NCT04197713 (STAR)	Phase Ib	Sequential treatment: adavosertib and olaparib	Patients with mutation in DDR genes or CCNE1 amplified PARPi pretreated (*n* = 13)	ORR = 3/12 (25%) SD = 5/12 (42%)	Hematologic (15%)	Yap et al. (81)

Aes, adverse effects; n, number of patients; PARPi, polyadenosine diphosphate–ribose polymerase inhibitor; HRD, homologous recombination deficiency; ORR, overall response rate; PFS, progression-free survival; O, olaparib; C, cediranib; A, adavosertib; DDR, deoxyribonucleic acid damage response.

### 4.1 The role of surgery

For a long time, surgery has been the cornerstone of advanced ovarian cancer treatment. Importantly, complete macroscopic resection is the most important prognostic factor in ovarian cancer. Residual disease might be associated with the presence of tumor subclones and intratumor heterogeneity and thus increase resistance to subsequent therapy. Of note, this statement remains true with the emergence of PARPi. Indeed, in the PAOLA-1 trial, *BRCA1/2*-mutated low-risk patients, i.e, patients with FIGO stage III disease who underwent macroscopic complete primary surgery, had a greater benefit of bevacizumab and olaparib maintenance therapy as compared to *BRCA1/2*-mutated higher-risk patients, i.e., patients with stage IV disease or with residual disease after upfront surgery or who received neoadjuvant chemotherapy (HR = 0.11 [95% CI = 0.03–0.31] and HR = 0.37 [95% CI = 0.23–0.59], respectively) (82). Moreover, in the case of oligoprogression under PARPi, surgery, as well as other local therapies and the continuation of PARPi, may have a role in overcoming PARPi resistance. Recently, a retrospective analysis from Gauduchon et al. showed that PARPi prolongation after local therapy for oligometastatic progression offers 11.5 months of PFS (83). Palluzi et al. also demonstrated a median prolongation of the treatment-free interval without platinum of 6 months and 10 months after surgery or stereotactic body radiotherapy, respectively, and PARPi continuation for patients with ovarian cancers experiencing oligoprogression under PARPi (84). Thus, surgery plays a major role in disease management.

### 4.2 The role of antiangiogenics to overcome PARP inhibitor resistance

PARP modulates the expression of genes involved in angiogenesis, with a particular impact on HIF-1*a*, which plays a significant role in tumor progression by orchestrating a comprehensive response to hypoxia (85). On the other hand, induction of hypoxia by antiangiogenic agents demonstrated a decrease in HR pathways’ efficiency through the downregulation of key genes such as *BRCA1/2* and *RAD51* (86). Thus, several preclinical data suggest the potential synergistic effect of PARPi in combination with antiangiogenic agents. In the first-line setting, the PAOLA-1 phase III clinical trial demonstrated a noteworthy improvement in PFS and OS of olaparib and bevacizumab first-line combination maintenance therapy versus bevacizumab alone in *BRCA1/2*-mutated patients and the HRD population (6, 87). Although the absence of an olaparib maintenance arm does not allow direct and strong conclusions, several data points suggest the potential benefit of the addition of bevacizumab to prolonge olaparib efficacy and therefore overcome PARPi resistance. First, a population-adjusted indirect treatment comparison was conducted that pooled patients from SOLO-1, assessing first-line olaparib maintenance therapy, and PAOLA-1 clinical trials. Results showed a numerical improvement in PFS in favor of bevacizumab and the olaparib arm as compared to olaparib alone in the *BRCA1/2*-mutated population (HR = 0.71 [95% CI = 0.45–1.09]) (88). Furthermore, while olaparib monotherapy as first-line maintenance seemed to have greater efficacy in *BRCA2*- compared to *BRCA1*-mutated patients in the SOLO1 trial (HR = 0.20 [95% CI = 0.10–0.38] and HR = 0.40 [95% CI = 0.29–0.56], respectively) (34), similar efficacy was observed in the PAOLA-1 trial with bevacizumab and olaparib combination (HR = 0.22 [95% CI = 0.09–0.54] and HR = 0.26 [95% CI = 0.16–0.41]) (29), highlighting the likely role of the combination in less sensitive *BRCA1*-mutated tumors. Moreover, in the high-risk (i.e., patients with residual disease after primary debulking surgery, neoadjuvant chemotherapy, or FIGO stage IV disease) *BRCA1/2*-mutated population from the PAOLA-1 trial, only 15% of patients experienced a progression within the first 15 months of olaparib and bevacizumab maintenance therapy (82). However, in the PRIMA trial, including high-risk patients comparable to the high-risk population from the PAOLA-1 trial, 34% of patients had progressed within the first 15 months of niraparib maintenance monotherapy (18). The same comparison can be done between *BRCA1/2*-mutated patients from the PAOLA-1 trial, including high- and lower-risk patients, and the *BRCA1/2*-mutated population from the ATHENA-mono trial, which assessed rucaparib in monotherapy as first-line maintenance. In the PAOLA-1 trial, a disease progression was observed for 12% of the patients within the first 15 months of combination maintenance therapy (87), while 23% experienced a disease progression with rucaparib monotherapy at the same time in the ATHENA-mono trial (17). However, these comparisons should be considered carefully, and the hypothesis must be confirmed with a prospective randomized trial. To this end, the ongoing NIRVANA and AGO-OVAR 28/ENGOT-ov57 trials assessing niraparib and bevacizumab first-line maintenance therapy versus niraparib alone address this question (89, 90). Also, the NIRVANA-R phase II single-arm study assessing the potential of combining niraparib and bevacizumab as maintenance therapy in patients with recurrent platinum-sensitive ovarian cancer who already received PARPi therapy (NCT 05183984) will provide more information about the role of bevacizumab in reversing PARPi resistance. In recurrent platinum-sensitive ovarian cancers, Mirza et al. reported a benefit in PFS with niraparib and bevacizumab compared to niraparib alone in intention-to-treat populations as well as in HRD and HRP (homologous recombination proficient). Patients (HR = 0.35 [95% CI = 0.21–0.57], HR = 0.38 [95% CI = 0.20–0.72], and HR = 0.40 [95% CI = 0.19–0.85], respectively) (91). In addition to bevacizumab, other antiangiogenics have been assessed in a recurrence setting, although less convincing. While the NRG-GY004 phase III trial assessing olaparib versus olaparib and cediranib versus chemotherapy in platinum-sensitive relapsed ovarian cancer patients did not show any difference between treatment arms in the whole population, subgroup analyses suggested the efficacy of the combination therapy in *BRCA1/2*-mutated patients. Indeed, the median PFS was 10.5 months, 18.0 months, and 12.7 months with chemotherapy, olaparib and cediranib, and olaparib alone, respectively, in patients with *BRCA1/2* mutant tumors (92). However, the EVOLVE study assessing olaparib and cediranib combination in HGSC patients after progression on PARP inhibitors was disappointing, with only 9% of ORR (three out of 34 patients). Of note, patients with *BRCA1/2* reversion mutations correlated with poor outcomes and did not benefit from the experimental combination (61), highlighting the restricted role of that combination in recurrence settings and suggesting the importance of using antiangiogenic with PARPi in earlier stages with the likely potential to delay PARPi resistance. Importantly, more translational research works are needed to understand how it might impact, if so, the occurrence of *BRCA1/2* reversion mutations.

### 4.3 Other agents that decrease homologous recombination pathway activity to overcome PARPi resistance

PIK3/AKT/mTOR pathway inhibition has also been associated with a decrease in the expression of *BRCA1/2* genes and impaired HR pathway activity, leading to a BRCAness profile (93, 94). Two phase I clinical trials investigating the efficacy of PI3KCA inhibitors in combination with olaparib showed promising results with an ORR of more than 30% (78). However, of the 10 partial responses, six occurred in *BRCA1/2* wild-type patients, while only three occurred in *BRCA1/2* mutation carriers, leaving a doubt about efficacy in *BRCA1/2* mutation carriers. Currently, a phase III randomized study, EPIK-O (NCT04729387), is undergoing assessment to evaluate the combination of olaparib and alpelisib versus standard chemotherapy in platinum-resistant ovarian cancer patients (95). Beyond antiangiogenic agents, targeting other protumorogenic pathways such as RAS/RAF/Mek pathways (96, 97) or targeting genes involved in epigenetics such as bromodomain-containing 4 (BET) (98) might also enhance PARPi efficacy. An ongoing phase I/II (NCT03162627) trial assessing olaparib and selumetinib, a MEK inhibitor, also includes an expansion cohort of PARPi-resistant ovarian cancers.

### 4.4 Targeting other DNA-damaging repair pathways to enhance PARP inhibitor sensitivity

Inhibition of the ataxia-telangiectasia and Rad-3 (ATR) kinases has garnered significant attention in the context of HGSC. This interest arises from the central role that ATR plays in the DNA damage response as well as in response to replication stress (99). ATR, along with its downstream effectors, checkpoint kinase 1 (CHK1) and WEE1-like protein kinase (WEE1), instigate cell cycle arrest and collaborate to rectify halted replication forks (100–102). Of note, some activity of ATR or WEE1 inhibitors in combination with PARPi in patients who are resistant to PARPi has been shown (80, 81, 103). More importantly, the underlying mechanism is mainly related to the restoration of replication fork stability. Nevertheless, the synergistic effect of ATR and PARPi has been observed across a wide range of PARPi and platinum-resistant models, including PDX models, that harbor different genetic alterations responsible for PARPi resistance, including the *BRCA1/2* reversion mutation (104).

### 4.5 Inhibition of the microhomology-mediated end-joining pathways to prevent *BRCA1/2* reversion mutation

MMEJ serves as a compensatory mechanism for repairing DSBs in the absence of HR. In addition, we previously showed that MMEJ pathways might drive *BRCA1/2* reversion mutations, which makes it a preferential target to overcome PARPi resistance and the occurrence of reversion mutations. Therefore, the inhibition of POLθ, an essential enzyme involved in MMEJ repair pathways, has gained specific interest (105, 106). The antibiotic novobiocin (NVB) is a specific inhibitor that binds to the ATPase activity of POLθ, leading to the inhibition of MMEJ repair. Both *in vitro* and *in vivo* evidence has demonstrated that NVB-mediated POLθ inhibition leads to synthetic lethality in HR-deficient tumor cells (107, 108). Recently, ART558, a small POLθ inhibitor, also induced DNA damage and synthetic lethality in *BRCA1/2* mutant tumor cells (109). However, the antitumor activity has been demonstrated in PARPi-resistant tumors driven by the defect of 53BP1/Shieldin complex, and efficacy in tumors that acquired *BRCA1/2* reversion mutation or the ability to delay reversion mutations still needs to be assessed. Also, the first oral-specific POLθ inhibitor, ART4215, is currently undergoing evaluation in a phase I/II trial, either alone or in combination with a PARPi (NCT04991480).

### 4.6 The role of immunotherapy to overcome PARP inhibitor resistance

Therapeutic strategies combining PARPi and immunotherapy have raised interest in *BRCA1/2*-mutated ovarian cancers. Indeed, *BRCA1/2*-mutated tumors exhibit a higher mutational load, an increased amount of neoantigens, tumor-infiltrating lymphocytes, and PD-L1 expression (110, 111). Single-cell analyses have recently revealed a higher immune infiltration by CD8+PD-L1+ T cells and a heightened co-occurrence of T cells, B cells, and antigen-presenting cells in *BRCA1/2*-mutated tumors, advocating for a more intricate collaboration of the immune system components compared to their wild-type counterparts (112). Interestingly, a recent pan-cancer research work also demonstrated an increase in T cells, natural killers, macrophages, and dendritic cells in *BRCA2*-mutated tumors, whereas *BRCA1* mutant tumors exhibited a higher presence of myeloid suppressive cells. Importantly, an enhanced OS with immune checkpoint inhibitors was noted in *BRCA2* mutation carriers compared to those with *BRCA1* mutations (113). In the prospective phase Ib/II TOPACIO/KEYNOTE-162 trial, the combination of niraparib with pembrolizumab was evaluated in patients with recurrent platinum-resistant ovarian cancers. The ORR was 18%, and the DCR reached 65%. However, ORR did not differ between patients harboring *BRCA1/2* mutations or not, and among eight patients who experienced long-term response, five were treated for a *BRCA1/2* wild-type tumor (114). The MEDIOLA phase II clinical trial investigated the combination of olaparib and durvalumab in *BRCA1/2*-mutated platinum-sensitive recurrent ovarian cancer patients. The ORR was 72%, and the median PFS was 11.1 months (115). However, the ARIEL2 trial assessing rucaparib in monotherapy in platinum-sensitive recurrent ovarian cancer patients showed a median PFS of 12.8 months and an ORR of 80% in the *BRCA1/2*-mutated population (116) This suggests that the addition of durvalumab might have only a limited impact, if any, on top of PARPi therapy. Of note, the JAVELIN PARP 100 phase III trial (NCT03642132), aiming to assess avelumab in combination with chemotherapy followed by maintenance avelumab and talazoparib in first-line ovarian cancer patients, closed for futility after interim analysis. The DUO-O phase III randomized trial (NCT03737643) assessing durvalumab in association with chemotherapy and bevacizumab and continuing with bevacizumab and olaparib as maintenance therapy in a first-line setting is ongoing to further answer these questions.

## 5 PARP inhibitor resistance: is there a place for a rechallenge?

**Figure 3:**
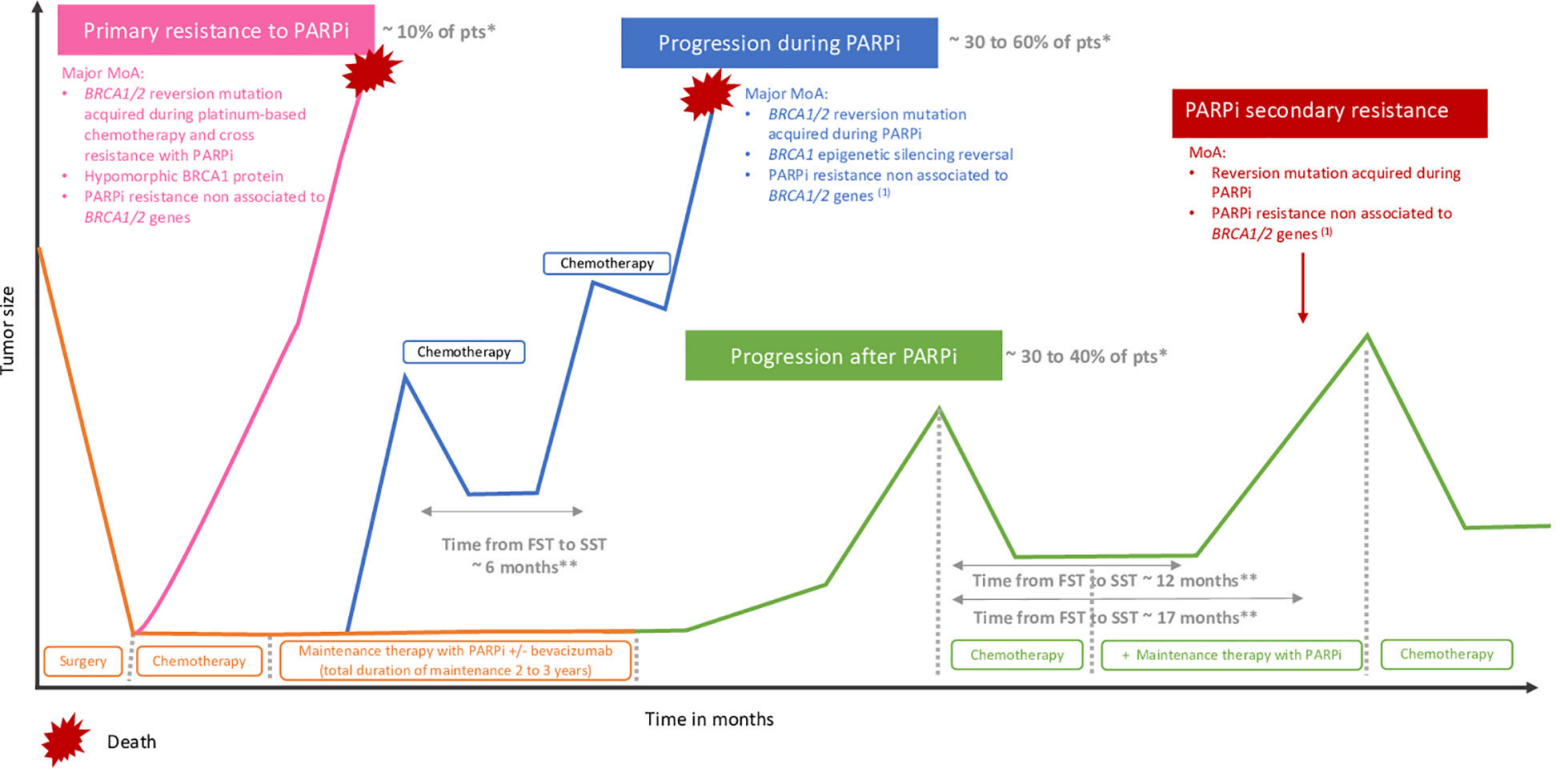
Primary resistance and progression during or after PARP inhibitors. (1) Upregulation of drug efflux pomps, PARP1 mutations, loss of DNA end protection, restoration of replication fork stability. *According to PAOLA-1, PRIMA, ATHENA-mono and VELIA. **According to PAOLA-1. MoA, mechanism of action; PAR, polyadenosine diphosphate-ribose polymerase inhibitors,; Pts, patients, FST, first subsequent therapy; SST, second subsequent therapy.

Despite the progression with PARPi, some patients might still benefit from a PARPi rechallenge. Aiming to characterize patients who will benefit from this therapeutic strategy, we need to achieve a clinical definition of PARPi resistance. To define PARP inhibitor primary and secondary resistance, we need to consider *BRCA1/2* mutation and HRD status, previous lines of chemotherapy, especially platinum-based chemotherapy, and PARPi treatment duration. Importantly, progression during or after PARPi maintenance therapy recently appeared to be an important prognostic factor to be considered. To this end, the OReO/ENGOT Ov-38 phase III clinical trial (117) is the only trial addressing the question of PARPi maintenance rechallenge. In this trial, patients who previously received PARPi maintenance in any line of treatment were randomized to receive olaparib maintenance rechallenge after platinum-based chemotherapy. Prior duration of PARPi exposure must be above 18 months following first-line chemotherapy or 12 months following a second or subsequent line of chemotherapy for the *BRCA1/2*-mutated cohort, and above 12 months and 6 months for the non-*BRCA1/2*-mutated cohort. The results demonstrated a benefit in favor of olaparib maintenance in both *BRCA1/2* mutant and wild-type cohorts (mPFS 5.3 months vs. 2.8 months, with and without olaparib, HR = 0.43 [95% CI = 0.26–0.71] in the *BRCA1/2* mutant cohort and 4.3 vs. 2.8 months HR = 0.57 [95% CI = 0.37–0.87] in the wild-type cohort). Although the results were statistically significant, the clinical benefit remains low. Moreover, no difference in time to subsequent therapy (TTST) was observed between treatment arms, although there was a trend in favor of the olaparib arm, and OS data are not mature. More recently, *post-hoc* analyses of the PAOLA-1 phase III clinical trial (6) also highlighted that patients progressing after or during olaparib might still benefit from PARPi rechallenge, with a median time to second subsequent therapy (SST) of 13.0 months and 6.0 months with and without PARPi rechallenge, respectively, in patients progressing during PARPi and of 18.5 months and 8.1 months, respectively, in patients who progressed after PARPi (118). However, patients selected for the PARPi rechallenge differed from patients who did not; these latter might have had less benefit from previous platinum-based chemotherapy and therefore were not offered a PARPi rechallenge. A forthcoming phase III randomized study will help in answering the question of PARPi rechallenge. Importantly, the greatest benefit of rechallenge would occur in patients who progressed after PARPi maintenance therapy, as suggested by *post-hoc* analysis from the PAOLA-1 trial showing a time from first subsequent therapy (FST) to SST of 6.1 months versus 11.4 months in patients who progressed under or after PARPi, respectively (119) (**Figure 3**). Specifically, a shorter time from platinum-based FST to SST was observed in patients progressing during olaparib as compared to patients whose disease progressed after olaparib or patients in the control arm (7.3 months vs. 12.0 months vs. 12.9 months, respectively) (119) (**Figure 3**). Consistent results were observed in the subgroup of patients receiving PARPi rechallenge after the first subsequent platinum-based chemotherapy (13.0 months vs. 18.5 months in patients progressing during and after PARPi first maintenance, respectively) (118). In the same line, retrospective analysis from the SOLO2 trial, assessing olaparib as maintenance therapy in *BRCA1/2* mutant platinum-sensitive relapse ovarian cancers, showed a significantly longer TTST in patients who previously received placebo as compared to patients who received olaparib as maintenance therapy (12.1 months vs. 6.9 months HR = 2.17 [95% CI = 1.47–3.19]) (43). In 2022, Oza et al. reported the overall survival results of the ARIEL4 study. Surprisingly, in the platinum-resistant subgroup, the authors demonstrated a better median PFS during the first subsequent therapy in patients who were randomized in the chemotherapy arm and therefore received paclitaxel before crossover to receive rucaparib compared to patients receiving rucaparib in the experimental arm (mPFS 7.3 months vs. 5.6 months, respectively). Interestingly, three out of four patients with platinum-resistant ovarian cancer treated by paclitaxel have a decrease of *BRCA1/2* reversion mutation, as suggested by the analysis of pre- and posttreatment samples (120). Furthermore, in the case of a rechallenge, we still need to assess which PARPi would be more efficient to overcome resistance. Importantly, forthcoming new PARPi, such as AZD9574, a highly selective inhibitor of PARP1 (121), might be of interest and is currently assessed in a phase I clinical trial (NCT05417594). These results suggest that the type of chemotherapy used before PARPi rechallenge may impact the subsequent response to PARPi, and that might be biologically explained through the interaction with the *BRCA1/2* reversion mutation. Clinical trials and translational research are strongly awaited to shed light on PARPi resistance and tailor therapeutic strategies to delay and overcome resistance.

## 6 Discussion

PARPi inhibitors have surely revolutionized the landscape of ovarian cancer treatment, but growing evidence highlights the need to delve further into molecular characterization in order to better select the patients who will benefit from the therapy. Indeed, locations and types of mutations of *BRCA1/2* genes generate a vastly different sensitivity to PARPi, with better outcomes in *BRCA2*-mutated patients (29, 122), and more specifically, a greater benefit in patients with mutations of exon 11 and in the DBD domain of *BRCA1*, and in RAD51-BD of the *BRCA2* gene. Interestingly, patients who harbor a mutation in the DBD domain of *BRCA2* seem to have excellent outcomes regardless of PARPi therapy. However, other large studies are pending to better refine the molecular characterization of *BRCA1/2*-mutated ovarian cancers and tailor therapeutic strategies. Moreover, most of the patients will experience relapse, with poorer outcomes for subsequent lines of treatment. In this review, we extensively studied the most known mechanism of resistance to PARPi so far: *BRCA1/2* reversion mutations. They are found in 40% of platinum-resistant (22, 53, 54) and less than 5% of platinum-sensitive ovarian cancers (23, 24, 54), as defined by the PFI, and are likely to confer PARPi primary resistance. *BRCA1/2* reversion mutations also occur under PARPi therapy, in 10% to 40% of cases, responsible for PARPi secondary resistance and occur mainly in the so-called hot spot regions such as the N-terminal or RAD51BD domains of *BRCA2* and the BRCT and RING domains of *BRCA1* (25, 26). The main objective should be to delay the occurrence of reversion mutations. Upfront surgery, whenever possible, and macroscopic complete resection remain the backbone of ovarian cancer treatment and confer a better PFS under PARPi maintenance therapy, likely by preventing the emergence of resistant tumor subclones under PARPi selective pressure. Some results also suggest the role of the addition of bevacizumab to PARPi first-line maintenance therapy to delay PARPi resistance in *BRCA1/2*-mutated patients, although the exact mechanism and impact on reversion mutation occurrence remain to be determined (82, 87). Paclitaxel might also act on reversion mutations and resensitize tumor cells to PARPi (120). After reversion mutation occurrence, the rechallenge of PARPi, alone or in combination with different targeted therapy encompassing PI3K/Akt/mTOR (78) or RAS/RAF/Mek/erk (96) pathway inhibitors, might still have a role, especially in patients who experienced progression after the completion of PARPi maintenance (119). Moreover, surgery, in the case of oligoprogression, to remove resistant tumor subclones, is of interest. We also need to keep in mind that, despite the overlap between PARPi and platinum salt resistance, some patients still derive a benefit from PARPi after resistance to platinum salts (117), and patients that progress despite PARPi might still benefit from platinum rechallenge, suggesting that some mechanisms of resistance or sensitivity might be drug-specific. Of note, Ceccaldi et al. reported that nucleotide excision repair (NER) pathway inactivation, accounting for 8% of HGSC from The Cancer Genome Atlas (TCGA), is associated with increased platinum sensitivity while not affecting PARPi sensitivity (51). More recently, an *in vitro* study demonstrated that the circular RNA circIGF1R_0001 increases the PARylation of *PARP1* and promotes platinum resistance while enhancing PARPi sensitivity (123). Some studies also describe the presence of multiple *BRCA1/2* reversion mutations and the variation of MAF of these mutations under PARPi pressure, suggesting that different subclones might impact sensitivity to PARPi or platinum salts during the course of the disease (27). Importantly, ctDNA appears as a very useful technology to depict intratumor heterogeneity, detect earlier, and monitor reversion mutations and resistant tumor subclones before and during PARPi therapy. However, how to tailor therapeutic strategies based on ctDNA remains to be adressed in prospective randomized trials to discuss the change of systemic therapy, the addition of antiangiogenic or targeted therapy, or local therapy in these patients.

## Author contributions

LC: Conceptualization, Writing – original draft, Writing – review & editing. BH: Conceptualization, Writing – original draft, Writing – review & editing. MT: Conceptualization, Writing – original draft, Writing – review & editing. IT: Supervision, Writing – review & editing. NC: Supervision, Writing – review & editing. OL: Supervision, Writing – review & editing. IR-C: Conceptualization, Supervision, Writing – review & editing.
